# Integrating 3D structural modelling and seismic interpretation to optimize hydrocarbon development in the Early Miocene Nukhul Formation, October Oil Field, Gulf of Suez, Egypt

**DOI:** 10.1038/s41598-025-29859-6

**Published:** 2026-03-01

**Authors:** Mostafa A. Khattab, Ahmed E. Radwan, Mohamed I. El-Anbaawy, Adel A. El-Tehiwy

**Affiliations:** 1 Geology department, Faculty of science, Cairo university, Giza, Egypt; 2 Faculty of Geography and Geology, Institute of Geological Sciences, Jagiellonian University, Kraków, Poland

**Keywords:** 3D structural modelling, October Oil Field, Hydrocarbon potentiality, Reservoir performance, Seismic survey, Reservoir–seal juxtapositions, Nukhul Formation, Gulf of Suez, Energy science and technology, Solid Earth sciences

## Abstract

This study presents an integrated 3D structural modelling workflow as a quantitative framework for applying advanced geological understanding to the spatial distribution of discrete and continuous reservoir properties of the underexplored, structurally complex Nukhul Formation in the October Oil Field. The updated 3D structural model was constructed by integrating high-resolution seismic interpretation with multidisciplinary subsurface datasets, including electric logs, detailed stratigraphic correlations, petrophysical evaluation, and production performance data from drilled wells. The current study specifically evaluates the structural controls on reservoir distribution and trapping styles within the field by combining 3D seismic interpretation with well-log and core-derived information. The refined model reveals a fault architecture dominated by a NNW–SSE (Clysmic) trend, with major faults dipping south-southwest (SSW) and horizons oriented northeast (NE), as determined from dipmeter data and seismic interpretation. The Nukhul reservoir is defined by a three-way dip closure, bounded by fault-dependent seals, where sealing capacity is governed by fault throw that juxtaposes permeable sand layers against impermeable lithologies. This configuration effectively inhibits cross-fault hydrocarbon migration, preserving attic accumulations. By integrating geological, geophysical, and petrophysical datasets, the new model significantly improves the delineation of attic targets, fault-bounded compartments, and reservoir–seal juxtapositions. Consequently, it provides refined well-placement recommendations, including high-potential attic infill drilling locations. Furthermore, the model establishes a technically robust basis for reservoir management and depletion planning, aiming to maximize recovery while minimizing water-handling risks. These outcomes demonstrate the value of incorporating new seismic and well datasets into legacy models and highlight their potential for reducing uncertainty in structurally complex syn-rift settings. Beyond the October Field, the updated structural modelling approach has broader implications for syn-rift petroleum systems in the Gulf of Suez and analogous rift basins, offering a predictive tool for fault-seal analysis, volumetric assessment, reservoir performance evaluation, and optimized drilling strategies.

## Introduction

The construction and refinement of reliable structural and static models are fundamental to hydrocarbon exploration and development. This process requires the integrated efforts of geology, geophysics, petrophysics, and reservoir engineering to synthesize diverse subsurface datasets into a coherent understanding of the reservoir^1^. Current petroleum industry research is increasingly focused on enhancing resource assessment accuracy and optimizing production efficiency through the development of high-precision structural models grounded in robust subsurface geological data^1–7^.

To refine structural model scenarios, it is essential to acquire and integrate diverse datasets, including high-resolution seismic surveys, well log interpretations, and stratigraphic correlations, supported by rigorous geological knowledge and interpretation workflows. Advanced modeling platforms are used to create updated and geologically consistent models by methodically connecting these datasets^1,8–11^.

Three-dimensional (3D) structural modelling has revolutionized subsurface imaging in the petroleum sector, enabling detailed delineation of reservoir architecture, fault network geometry, stratigraphic compartments, and hydrocarbon trapping mechanisms^1,2^. A precise 3D structural framework not only supports volumetric estimations and reduces drilling risk but also identifies high-potential zones for future exploration and field development^8,12,13^. Nevertheless, despite the proliferation of advanced modelling software, achieving modelling accuracy remains a persistent challenge with practical consequences for hydrocarbon recovery strategies and economic feasibility.

In the Gulf of Suez, the October Field has been the subject of multiple petroleum system investigations, including carbonate reservoir studies in the Radwany Formation^14,15^, reservoir characterization of the Nubian Sandstone^16,17,48^, and petrophysical evaluations of the Asl Formation^18^. However, the Early Miocene Nukhul Formation, a stratigraphic interval with potential reservoir quality, has received comparatively limited attention. Previous 3D structural modelling efforts, notably by GUPCO in collaboration with Amoco (1992)^19^ and later with BP (2012)^19^, focused primarily on the Miocene section but were constrained by the limited seismic and well datasets available at the time.

With the acquisition of new high-quality seismic data and recently drilled wells, there is now an unprecedented opportunity to revisit and enhance the structural understanding of the Nukhul Formation. This study presents a novel, integrated 3D structural modelling approach applied to the October Field with the objective of appraising and developing the hydrocarbon potential of the Nukhul Formation, thereby providing a more accurate framework for static modelling, facies distribution analysis, and optimized field depletion planning.

While the Gulf of Suez Basin is a mature hydrocarbon province, the structural complexity introduced by fault reactivation, block tilting, and syn-rift tectonics has left certain stratigraphic units—such as the Nukhul Formation—underexplored. This research addresses the knowledge gap by delivering the first high-resolution, data-rich 3D structural model for the Nukhul Formation in the October Field, integrating legacy datasets with newly acquired seismic and well data. The novelty lies in combining multi-scale structural interpretation with updated geological constraints to produce a model that is both geologically realistic and directly applicable for reservoir simulation and production optimization.

## Geological setting

**Fig. 1:**
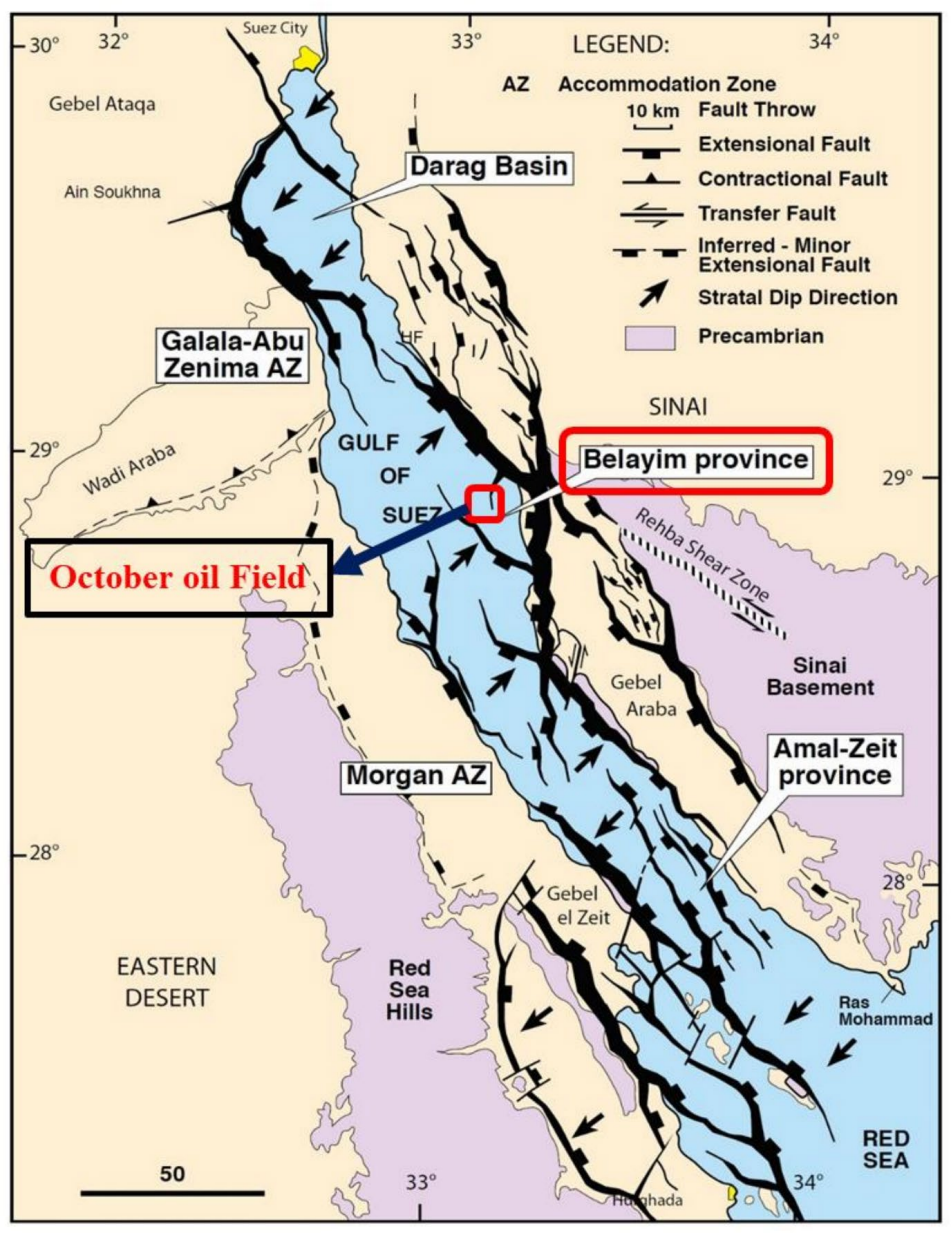
Location map of the studied October oil field within the Belayim province, Gulf of Suez^20^.

**Fig. 2:**
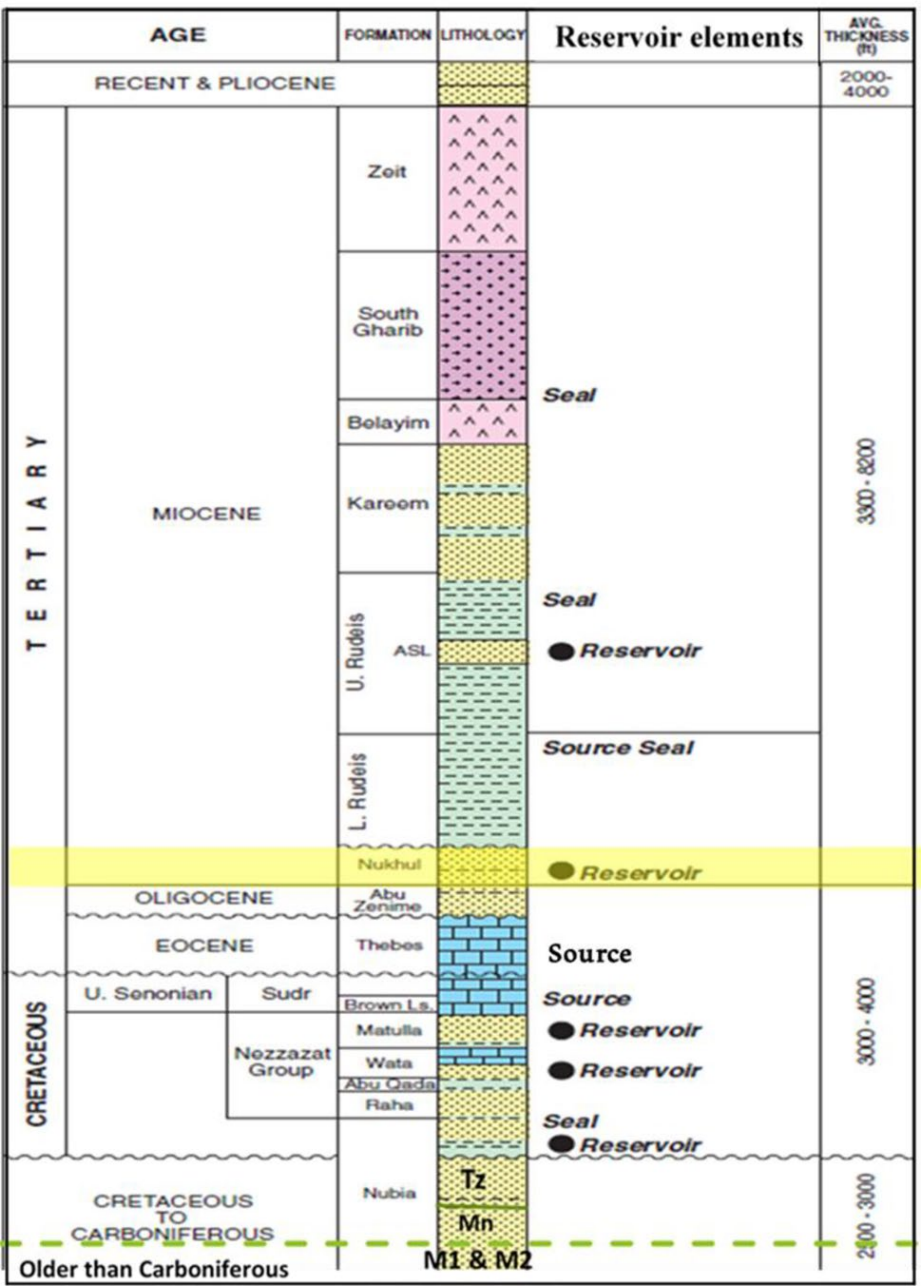
The stratigraphic geological column emphasizing the Nukhul Formation, showing the generalized lithologies and thicknesses in of the October oil Field; in addition to representing the whole section for Miocene & Pre-Miocene (After GUPCO, 2012^19^).

The studied field is one of the Egypt’s oldest petroleum-producing regions (Fig. 1). After the Morgan and Belayim Marine oil fields, it is the third-largest oil field in the Gulf of Suez. This world-class massive oil field extending over approximately 30 km and consists of nine major fault blocks^43^. The Gulf of Suez’s geological sequence extends from the Precambrian to the Holocene. Figure 2 depicts the October Oil Field’s stratigraphic column.

**Fig. 3:**
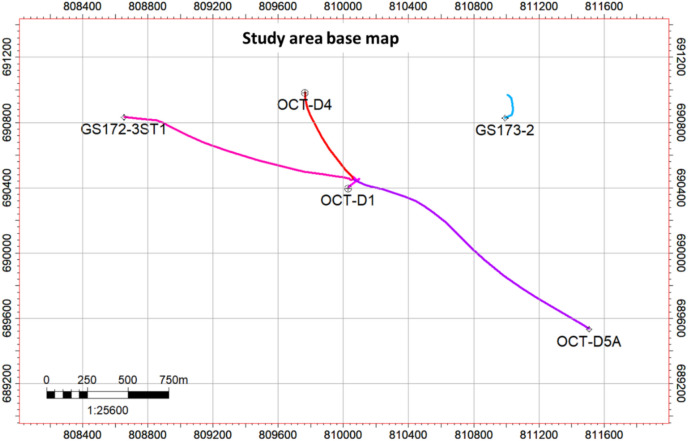
Base map shows the distributed studied wells.

The principal reservoirs, from the lowermost part of the geological column (Fig. 2) upward, are the Nubia Sandstone, Nezazzat Group, Nukhul Formation, Upper Rudeis Formation, and Belayim Formation. The field is located on a NW-trending fault block in the province of Belayim. Normal faults that dip westward around pre-Miocene fault blocks that dip northeast^21,40,47^. The Nezazzat Mountain Range, which outcrops to the south along the Sinai Coast for roughly 20 km (12 mi), is thought to be an offshore, down-failed continuation of the October Oil Field trend^22^. The field’s structural makeup is a series of rotating fault blocks that are common in rift basins all over the planet. Thus, like many other fault-block fields, the October Oil Field is a sequence of rotating fault blocks typical of the Gulf of Suez Rift. However, the October Oil Field is structurally more complex than it might initially appear^23^. Figure 3 illustrates the study wells, serving as examples of those used in the structural model update.

There are several different reservoir accumulations, with the GS173-2 well showing the most notable oil–water contact at − 10,496 feet. In the October Oil Field, oil gravities range from 14° to 39° API (Amoco report, 1987). Hydrocarbon generation is primarily associated with two major source rock units—the Campanian Brown Limestone and the Eocene Thebes Formation (Radwany Formation)—with additional geochemical evidence suggesting contribution from the Lower Rudeis source rock to the October D area (GUPCO, internal report, 2024^19^). Cumulative production from the October D area’s Nukhul Sandstone reservoir is approximately 18.5 MMBO (million barrels of oil) (GUPCO, internal report, 2024^19^). The Nukhul Formation, of Lower Miocene age, corresponds to the T10 unconformity surface. This formation unconformably overlies the pre-Miocene sequence, which begins with the Thebes Formation (T00 unconformity surface) and terminates at the granitic basement. It is overlain by the Miocene succession, ranging upward from the Rudeis Formation to the Zeit Formation (Fig. 2). Typical reservoir parameters for the Nukhul Sandstone, based on cored well data, are porosity of 13–19%, permeability of 340–640 mD, and net pay thickness of 70–180 ft.

## Methodology

### Materials

20 seismic lines that covered the October Oil Field and five stratigraphic and structurally controlling wells—GS172-3ST1, OCT-D4, OCT-D1, GS173-2, and OCT-D5A—were made available for this study by EGPC via GUPCO (Fig. 3). A set of E-logs, which include Caliper logs (CALI), Gamma Ray logs (GR), Resistivity logs (RD), Density logs (RHOB), Neutron logs (NPHI), Sonic logs (DT), Dip-meter data, Core data, Paleontological data, well test results, and production data, as well as the chosen seismic lines that span the study area, are among the materials. Microsoft Office Excel, LAS Tool, Canvas X 8.0, Techlog Software, Microsoft Office, Schlumberger chart books 2005 & 2006, and Petrel software are among the software-based processing and manual computations that form the basis of this study.

### Workflow

**Fig. 4:**
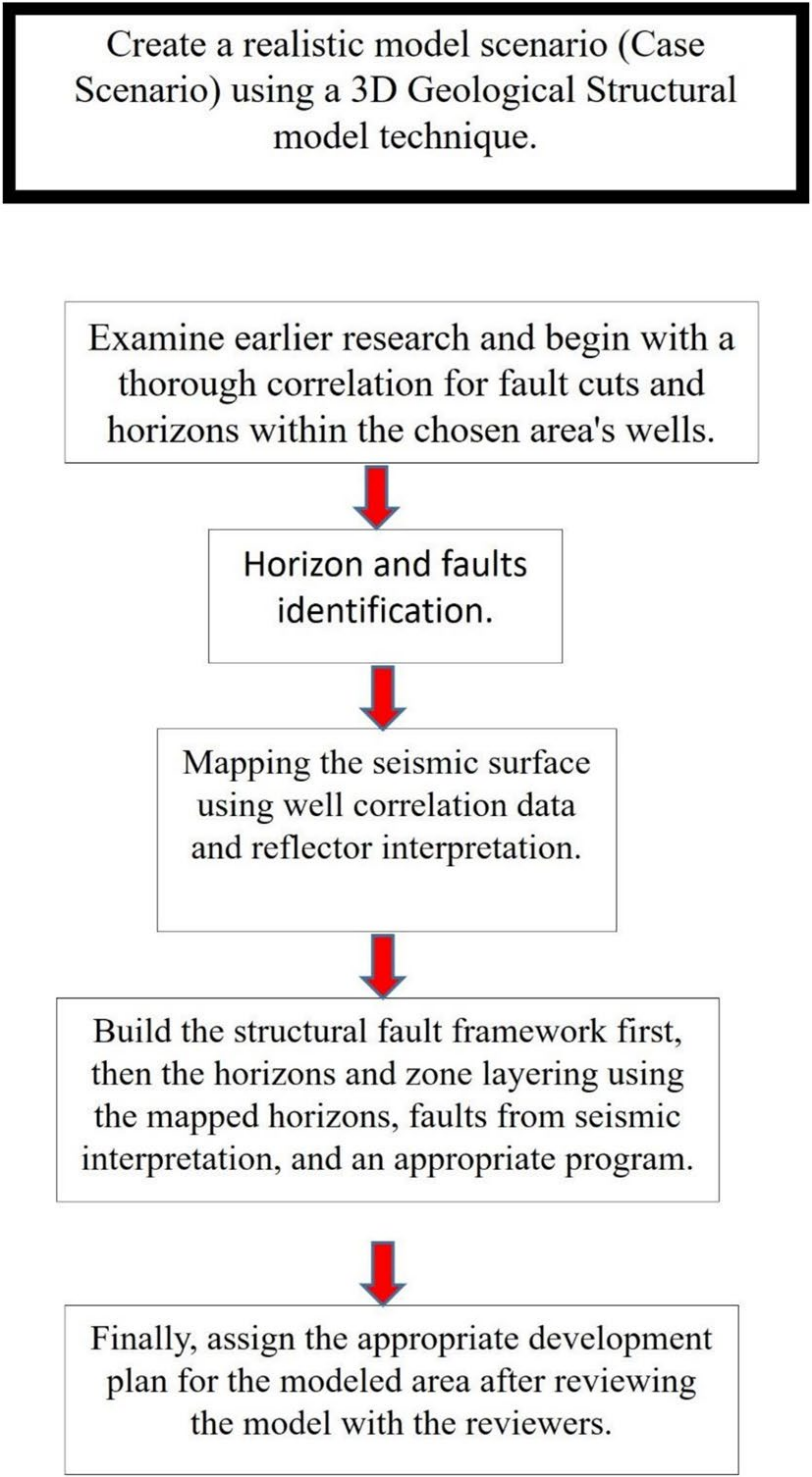
Flow chart of the used integrated modelling work.

The workflow for this study addresses the main challenges of the structural area and the approaches used to overcome these obstacles by utilizing the available data, as shown in Fig. 4. Well data (including detailed correlation using ditch-cutting descriptions and E-logs, dynamic data such as production and pressure, and other related datasets), outcrop interpretation data as analogues for the subsurface, geophysical or seismic data sections, and historical interpretations (including previous models and their conceptual frameworks) were all incorporated. The main primary challenge in developing the structural model was to synthesize and correlate all available data to produce a clear structural framework that facilitates the identification of new exploration opportunities and improves understanding of the area of interest. Multi-source datasets were integrated into the 3D model. The workflow to modify the structural model consisted of the following steps:

- Collecting and reviewing previous work and datasets relevant to this study to establish a solid foundation of knowledge and avoid duplicating earlier efforts. In addition to study the geological background information, surface data such as outcrops were used to generate a conceptual model that guided the software-based modelling process.
- Detailed correlation of the selected wells: Using geological cutting descriptions and E-logs, formation boundaries and fault cuts were first identified.
- Multiple cross sections were created along the wells to better understand the structural configuration. The integration of these correlations with cross-section interpretations was carried out, and the well picks and fault cuts were then connected with seismic reflectors to link horizons and faults. Dynamic flow data—including well flow rates, transient pressure/height measurements, and fluid composition data—were also incorporated. Such datasets help to identify faults, especially in regions distant from wells, by indicating the connectivity of subsurface reservoir rocks, enabling the develop of a structure contour map.
- Seismic interpretation involved integrating well picks with seismic reflectors to delineate horizons and faults, thereby updating the structural model within the software environment.
- The final interpreted seismic horizons were used to update the structural model, incorporating all relevant horizons and geological zones^1,8,24–26^. The processing phases flowchart is shown in Fig. 4. These illustrating all the procedures undertaken to modify and update the structural model to meet the development plan objectives
- The last stage involved combining data and interpretations from multiple disciplines to produce a coherent, integrated 3D structural model based on geological and seismic datasets. This model was subsequently used to identify new development opportunities for field development planning.

### Methods

The logging data were checked for quality before interpretation. To comprehend the complex geometry, an integrated study utilizing interdisciplinary datasets (such as 3D seismic and wireline logs) was carried out according to Ali et al.^8^. To put the study procedure into practice, additional data that was accessible, including wellheads, formation tops, and check shots, was gathered, examined, and combined following the study workflow (Fig. 4). To construct an accurate geological model, several seismic interpretation techniques, including seismic attributes, seismic manipulation (including structural smoothing and instantaneous phase), as well as conventional interpretation methods, were applied throughout the workflow^27^. In the surveys under study, seismic–well connections were conducted using wells having density, sonic velocity, and Vertical Seismic Profile (VSP) data. This produced a well time–depth relationship (TDR) between the seismic volumes and the related wells. The following formulas were used to determine acoustic impedance during the seismic–well tie procedure by multiplying density and sonic logs^28^:

1 $${\mathrm{z}} = \rho {\mathrm{v}}$$

2 $${\text{v }} = \, \surd \left( {{\mathrm{E}}/\rho } \right)$$

where the z is acoustic impendence, ρ is the density, v is sound wave velocity, E is elastic modulus.

A synthetic seismogram was constructed by convolving the derived acoustic impedance over various formation densities and sonic velocities with a specified wavelet. Formations within the seismic volume under study have been selected using the seismograms produced for the wells under investigation. Faults were interpreted using the 3D seismic volume. To enhance fault interpretation (e.g., edge detection), structural seismic volume features like variance, anti-tracking, and structural smoothing were used.

A boundary polygon was generated by digitizing the survey map in order to identify the region of interest during horizon mapping. All of the data inside this boundary was then modeled and mapped. The interpreted faults and horizon were utilized as inputs for generating structural maps which had been enclosed by the polygon. High-density seismic lines and a convergent interpolation algorithm were used to produce these structural maps.

Using these surfaces as inputs, a velocity model has been developed, and this velocity model was then used for depth conversion. The surfaces covered the entire velocity and determined the value at each x y coordinate. The velocity model used the following equation According to Al-Chalabi^29^:

3 $$v = vo + kZ$$

The interpretation was done using 3D PSDM (Pre-Stack Depth Migration) data. The structural model was updated to include the faults and horizons that were interpreted from this dataset. Following that, structural depth maps and the structural grid were created.

Uncertainty treatments and formal QC procedures were integrated throughout the workflow to improve methodological rigor: well ties were anchored using check shot- and VSP-calibrated time-depth relationships, and seismic-to-well mis-tie statistics (mean residual, standard deviation, and RMS error) were computed. In order to prevent the introduction of non-physical deformations where systematic timing offsets were present, we used linear drift corrections and, in localized intervals only, bounded stretch/squeeze adjustments (with constrained dynamic-time-warping or limited percent-stretch tolerance). In order to retain fault edges while reducing noise, dip-guided structural smoothing was performed to the seismic volumes. restricted cubic-spline (or restricted kriging) interpolation was used to interpolate missing or weak horizon segments, subject to thickness and trend limitations. Cross-section checks and the enforcement of fault-cut linkages generated from variance/anti-tracking features were used to validate fault and horizon consistency. Lastly, depth-conversion and velocity-model dependability were evaluated by comparing depth-converted surfaces to recorded well tops, reporting depth residuals, and iteratively modifying model parameters (e.g., velocity gradient, *k*) until residuals satisfied the specified QC criteria. In order to guarantee reproducibility and to connect the workflow with current best practices, these techniques were assembled under a formal "Uncertainty and Model Validation" component.

## Results

### Seismic interpretation

**Fig. 5:**
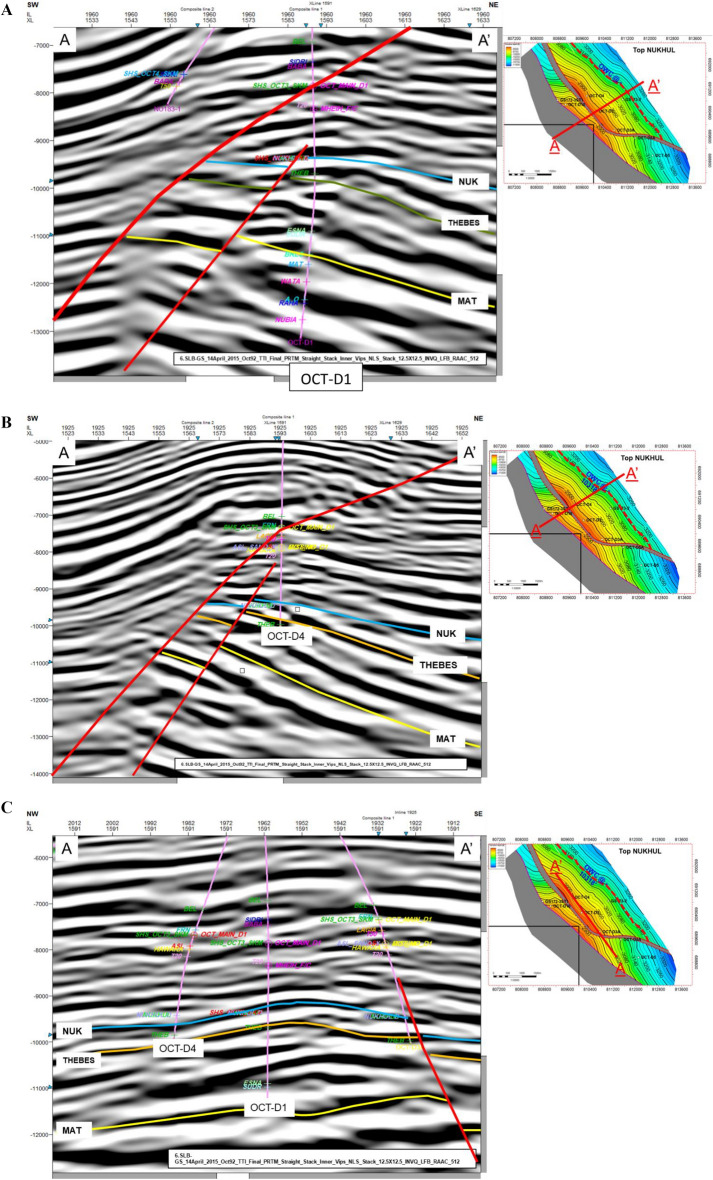
(**A**) Seismic dip line cross-section along the OCT-D1 well, illustrating the stratigraphy and structural framework of the study area. The location of the section is shown on the accompanying structure contour map. (**B**) Dip line cross-section along the OCT-D4 well, showing the stratigraphy and structure of the study area. (**C**) Seismic strike line cross-section along the OCT-D4 and OCT-D1 wells.

Providing accurate and comprehensive evidence about the structural and stratigraphic features of the seismic potential is the main objective of seismic interpretation. In order to guarantee precise structural analysis, the traditional interpretation method was used^41,46,50^. Variations in acoustic impedance inside subterranean rocks with varying physical characteristics are what create reflections^30^. Given that the data used were depth migrated, and the velocity model was calibrated to wells, there was no need for well-to-seismic tying^8,39,45,49^. The reflection coefficient at the interface was used to model horizons across the whole dynamic range of the reflectivity data. Horizons and flaws were carefully chosen after the previously extracted properties were scanned. The tying process was used to analyse all of the horizons in the seismic sections. The general seismic section across the wells of interest, showing the stratigraphy and structure of the research area, is shown in Fig. 5A–C. The maps confirm the existence and continuation of the faults based on the newly acquired seismic data and the incorporation of borehole data.

### Structural and stratigraphic correlation

**Fig. 6:**
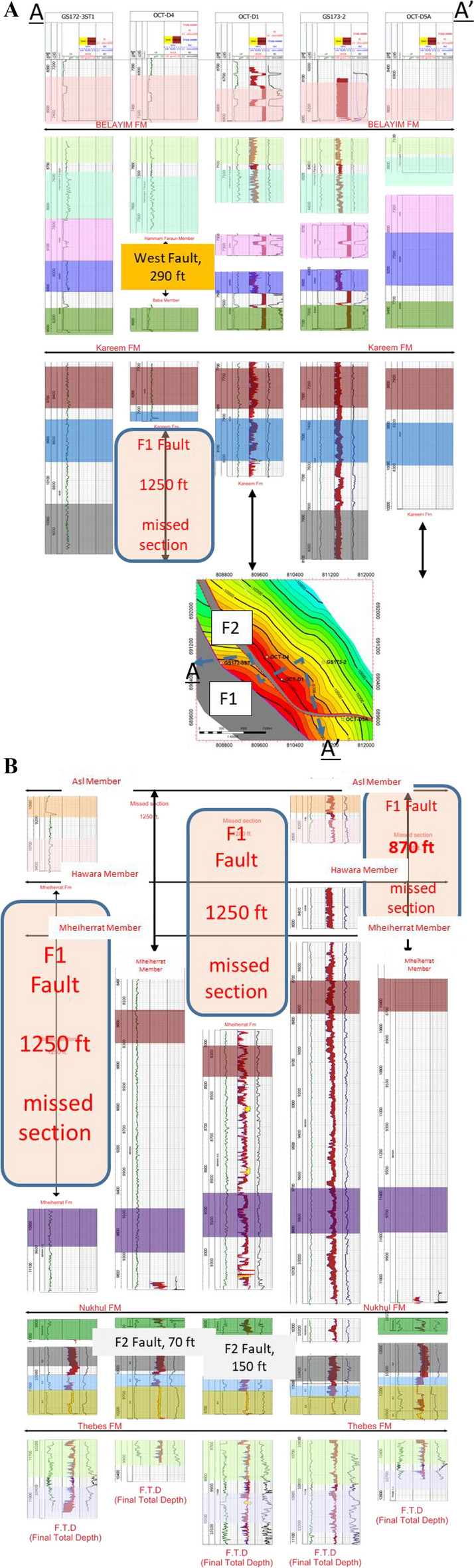
(**A**) Correlation line A-A', illustrating the structural and stratigraphic framework that supports the proposed structural model. (**B**) The second part of correlation along the same line of section to show the stratigraphic and structural framework supporting the suggested structural model.

The available data used for the correlation are the five wells. The wells are distributed across the area of interest to cover as wide an area as possible, providing more accurate and realistic results. This interpretation is based on the integration between the regional understanding of the basin and oil field structural setting (e.g.,^14,15,31^). The fundamental skeleton of the structural model was updated in conjunction with the present and previous research, which included seismic interpretation, well correlation, and dynamic data. The structural correlation is thoroughly examined using available data. Several markers were interpreted between the five wells at the Miocene section level (Fig. 6A,B). The main formation boundaries are the Belayim, Kareem, Asl, Hawara, Mheiherrat, Nukhul, and Thebes formations, as well as all members such as Hammam Faraun, Feiran, Sidri, and Baba within the Belayim Formation. In addition, some markers occur within each formation and member, with these units color-coded in all wells to make it easier to detect formation boundaries and, at the same time, to identify missed units. The selected wells are representative of the structure and stratigraphy of the study area; the wells contain a complete set of data, including E-logs and ditch-cutting samples. Three of these wells have core samples (GS173-2, OCT-D4, and OCT-D5A wells). All of these data are highly useful in modifying and updating the structural model. By precisely identifying the missing geological sections at each level throughout the stratigraphic column (Fig. 6A,B) and its extension throughout the region to integrate all linked wells and create a dependable structural framework, correlation was constructed to comprehend fault heave or throw.

- The first well in this correlation is GS172-3ST1, drilled to the northwest of the study area as shown in the base map (Fig. 3). This well reached the final total depth (FTD) in the Thebes Formation and targeted the Nukhul Sandstone reservoir. The well intersected the main fault (F1) from the lower part of the Kareem Formation to the lower part of Mheiherrat Member, missing about 1250 ft of section. The missing geological record is correlated with the GS173-2 well as a reference or type log in this drilled section. Drilling began in the South Gharib Formation, followed by Belayim and Kareem formations, before intersecting the main fault (F1). As a result, the well missed the remainder of the Kareem Formation, the Asl Member, the Hawara Member, and the top and middle parts of the Mheiherrat Member. Finally, it drilled the rest of the Mheiherrat Member and the Nukhul Formation until it reached the Thebes Formation, where drilling stopped.
- The second well is OCT-D4, drilled southeast of the GS172-3ST1 well. The well was completed in the Thebes Formation, targeting the Nukhul Sandstone reservoir. There are three missing sections in this well. The first is within the Belayim Formation, from the Hammam Faraun Member to the Baba Member, missing 290 ft due to a west fault from the main fault (F1). This west fault is not of interest in this study because it lies west of the main fault, meaning its block is downthrown from the main block, indicating no possible opportunity. This fault correlates with the GS172-3ST1, OCT-D1, and GS173-2 wells. The second missing section relates to the main fault (F1), with about 1,250 ft missing from the middle part of the Kareem Formation to the lower part of the Hawara Member. This missing section is correlated with the GS173-2 well as a reference log. Drilling started in the South Gharib Formation, then passed through the Belayim and Kareem formations before hitting the main fault (F1), which resulted in missing the remainder of the Kareem Formation, the Asl Member, the Hawara Member, and the top and middle parts of the Mheiherrat Member. Finally, it drilled the rest of the Mheiherrat Member and the Nukhul Formation until it reached the Thebes Formation. The third missing section is within the Nukhul Formation (F2 fault), missing about 70 ft. The Nukhul Formation can be divided into four members, from K1 to K4. These members are useful in detecting reservoirs and missing sections. In this case, the well missed the basal part of the K2 member and the top part of the K3 member (Fig. 6). The correlated wells are GS172-3ST1, GS173-2, and OCT-D5A.
- The third well is OCT-D1, drilled south of the OCT-D4 well and southwest of the GS172-3ST1 well. The well was completed into the Nubia Formation to test Pre-Miocene target reservoirs, while the main target was the Nukhul Formation, which is the focus of this study. Therefore, the study stopped at the Thebes Formation and did not include the remainder of the well trajectory. The well intersected two faults, creating two missing sections. The first is the main fault (F1), missing about 1,250 ft from the middle part of the Kareem Formation to the upper part of the Mheiherrat Member. This fault resulted in the loss of the lower part of the Kareem Formation, the Asl and Hawara members, and the upper part of the Mheiherrat Member. The second missing section, about 150 ft, is associated with the F2 fault and extends from the K1 member of the Nukhul Formation to the top part of the K3 member (Fig. 6A,B). The correlated wells are GS172-3ST1, GS173-2, and OCT-D5A.
- The fourth well is GS173-2, drilled in the lowest down-dip position of the main block and eastward compared to all other wells in the study area. The well was completed in the Nubia Formation to test Pre-Miocene targets, while the main target was the Nukhul Formation. This is the main focus of the study, so analysis stopped at the Thebes Formation, similar to OCT-D1. This well had no missing sections because it was drilled vertically without directional planning. It is the most down-dip well of the main block, located away from block faults. Drilling began in the South Gharib Formation, then passed through the Belayim, Kareem, Asl, Hawara, Mheiherrat, and Nukhul formations of the Miocene section, before continuing into the Pre-Miocene section, including the Thebes, Esna, Sudr, and Brown Limestone formations, the Nezazzat Group, and finally the Nubia Formation.
- The fifth well is OCT-D5A, drilled in the southernmost part of the study area. The well was completed in the Thebes Formation, targeting the Nukhul Sandstone reservoir. It intersected one fault: the main fault (F1), missing about 870 ft from the lower part of the Kareem Formation to the upper part of the Mheiherrat Member. This missing section includes the lower part of the Kareem Formation, the Asl and Hawara members, and the upper part of the Mheiherrat Member. The correlated well is GS173-2.

### The geological structural contour maps

Structure contour maps are essential and critical to initiating any depletion plan. The purpose of structure contour maps is to identify appraisal, exploration, and development opportunities. All developed opportunities are built based on fault positions, attic locations, reservoir extent, and compartmentalization within any given study area. The current study is based on modifying and updating the existing structure contour maps with a new structural model scenario derived from the integration of all available data, such as E-log data, paleontological data, ditch cutting sample descriptions, updated seismic interpretation and manipulation, detailed structural and stratigraphic correlation, and updated petrophysical evaluation to support volumetric calculations, which will support the identification of opportunities.

**Fig. 7:**
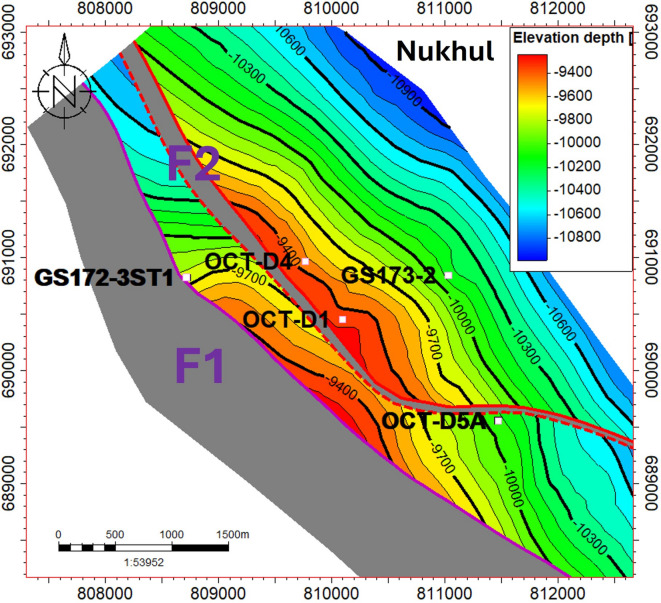
Structure contour map on the Nukhul level illustrating the two main structural compartments.

The developed model shows that the studied area is mainly controlled by three faults, but the model focuses on only two faults, F1 and F2, because these two faults have the greatest impact on any future opportunity, as shown in (Fig. 9). The structure contour map in (Fig. 7) shows two different compartments at the Nukhul reservoir level. Any structural contour map may generate an unlimited number of ideas for novel prospects, for instance, development or injection wells, to support the area’s production and well pressure for better reservoir management when it is based on sound foundations and combined data.

The map in (Fig. 7) confirms the existence of numerous opportunities that will be shown and discussed next in the conclusions and summary section. Field development has faced various difficulties in reservoir engineering and exploration following the drilling of new wells and the implementation of development plans, even though the map’s accuracy is dependent on the most recent data available. Over time, the collection of reservoir data, seismic data, and new wells, including approximately 12 newly drilled wells with complete logging data after 1992, along with pressure and dynamic data, provided an outstanding opportunity to update and adjust the structure model using this newly acquired information. Numerous questions that have lately emerged during reservoir exploitation have been answered according to the new data.

**Fig. 8:**
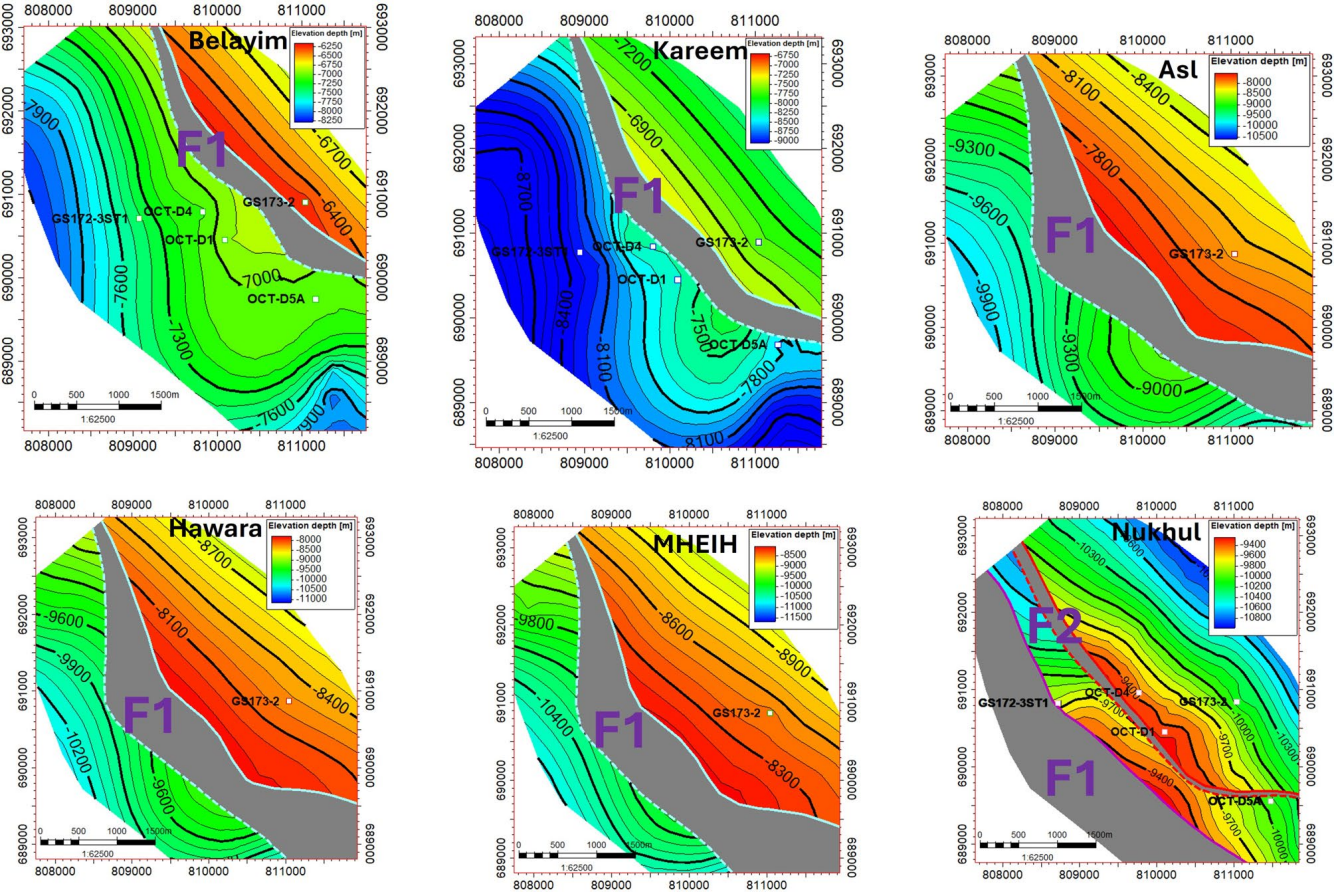
New structure contour maps for the Miocene section were generated from the updated structural model to define block extensions. These maps identified the F1 and F2 faults as the primary controlling structures.

**Fig. 9:**
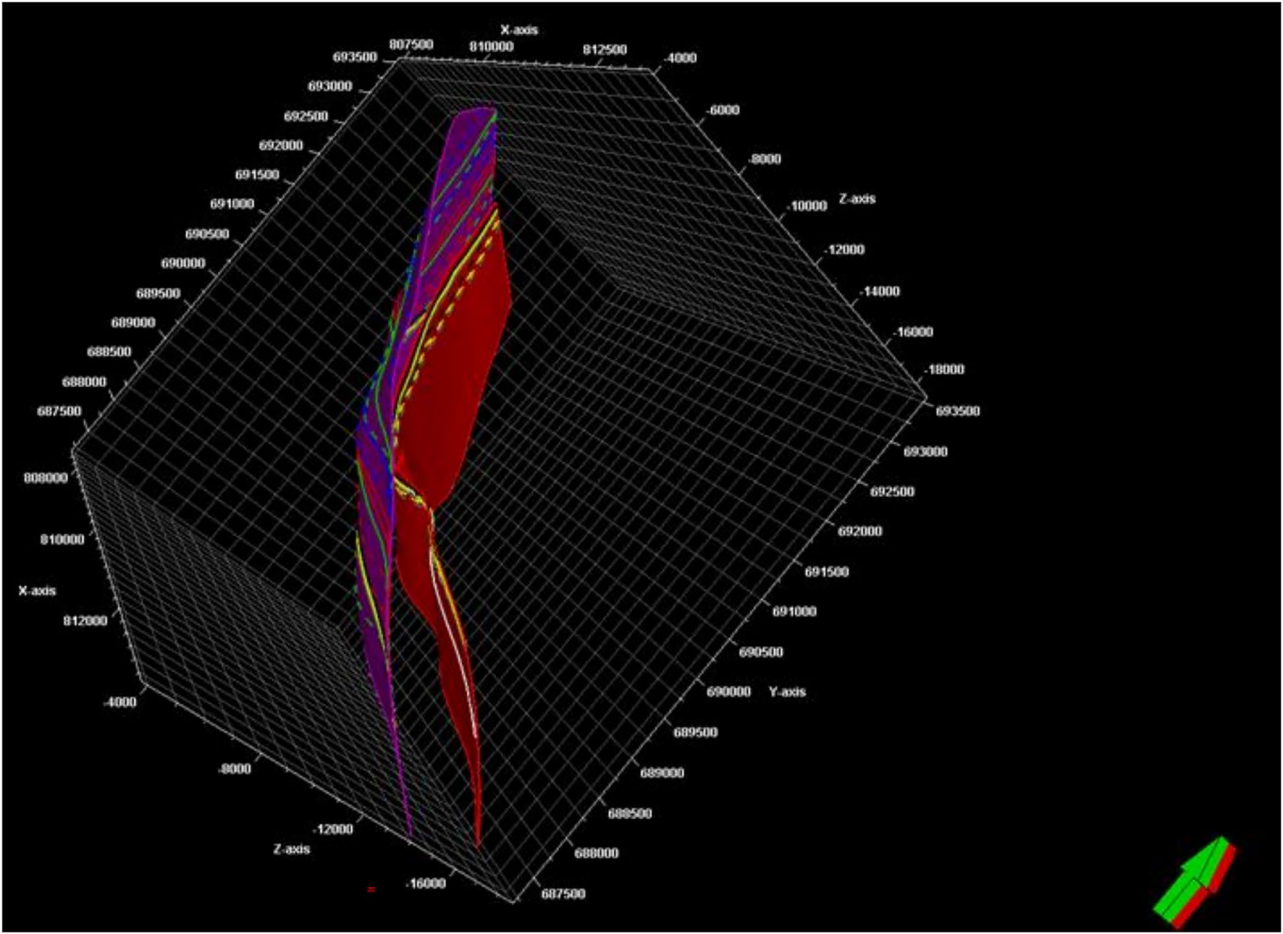
The final 3D structural model, incorporating the two key faults F1 (pink) and F2 (red). These faults, identified on structure contour maps, critically influence future exploration opportunities.

The structural model was initiated and finalized along the Miocene section for all formation members and zones to ensure realism and to show the continuity and consistency of the interpretation as shown in (Fig. 8). The structure type and its extent along the Miocene segment are well depicted by the model. The primary contributing faults in the region of interest, the F1 and F2 faults, are depicted on the structural contour maps in (Figs. 7, 8, and 9).

### Geological cross sections

**Fig. 10:**
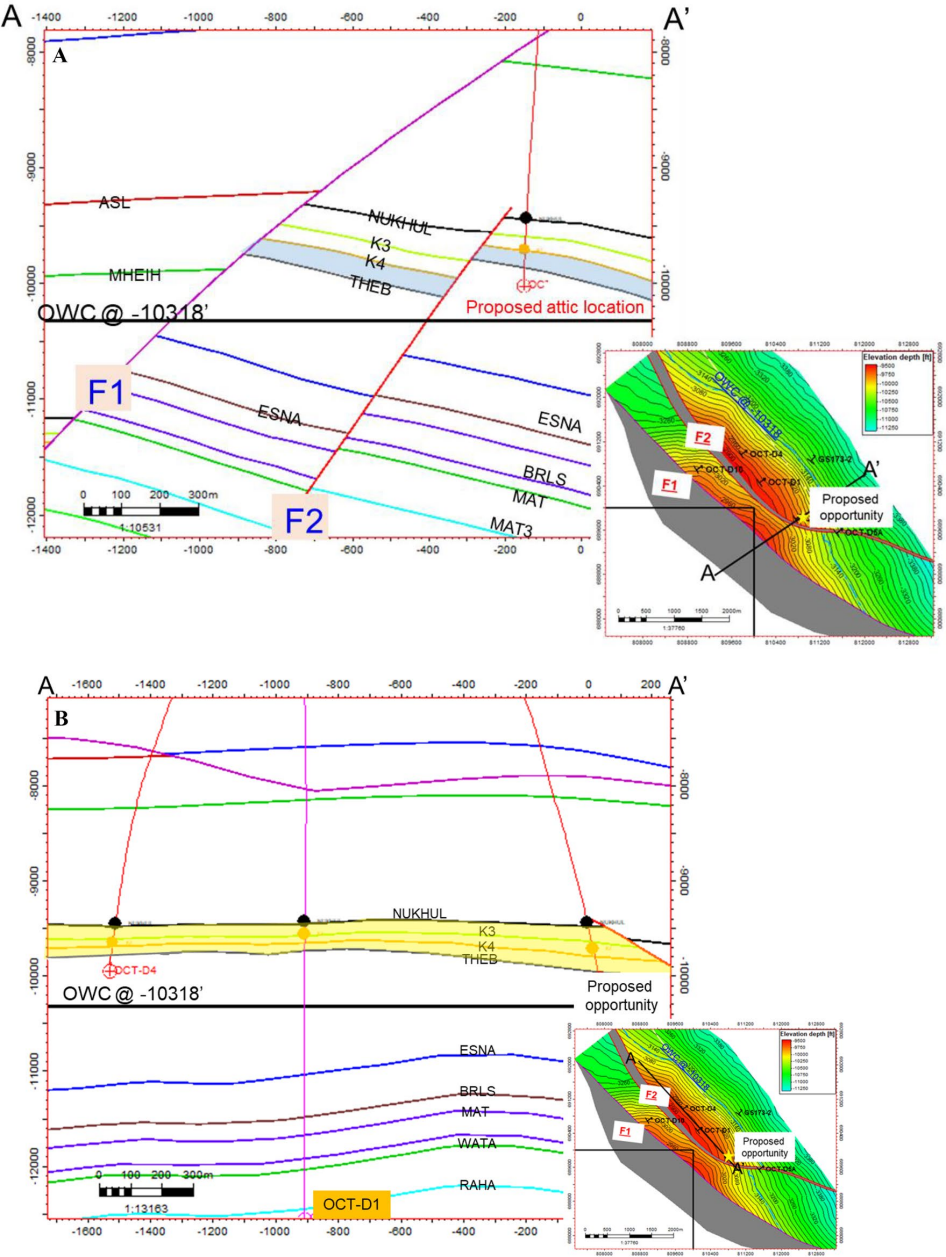
(**A**) Dip line cross-section through the proposed opportunity, illustrating the impact of the updated structural model. (**B**) Strike line section (SLS) along wells OCT-D4 and OCT-D1, showing the structural relationships.

Following the completion and evaluation of the structural model, there was a need to show the different geological cross sections, particularly reflect the extension of the block borders and faults, as seen in Fig. 10A and B. To effectively depict the structure, the geological cross sections are based on various section lines, including strike and dip lines. The relationship between the well formation boundaries that support the structural model scenario reflects this concept. Based on the updated structural model, there was an idea for a well opportunity as an attic well. Figure 10A illustrates the dip line of section (DLS) along the proposed opportunity to show its validity and proposed location. This line of section is very supportive and clear, especially as it shows the opportunity location, the entire stratigraphic section (Miocene and Pre-Miocene), the full formation juxtapositions (the relationships between all structurally proposed blocks), and the last updated oil–water contact.

A strike line of section (SLS) was constructed along the OCT-D4, OCT-D1 wells, and the proposed opportunity wells to show the stratigraphic sequence for the entire stratigraphic column (Miocene and Pre-Miocene sections), its relationships, and structure along the study area, as shown in (Fig. 10B). This line of section was needed to show the relationship between the wells to further validate the proposed well opportunity. The two lines of section, along with other geological cross sections in the background, support the reconstructed structural maps of the October Oil Field.

### Fault seal analysis

**Fig. 11:**
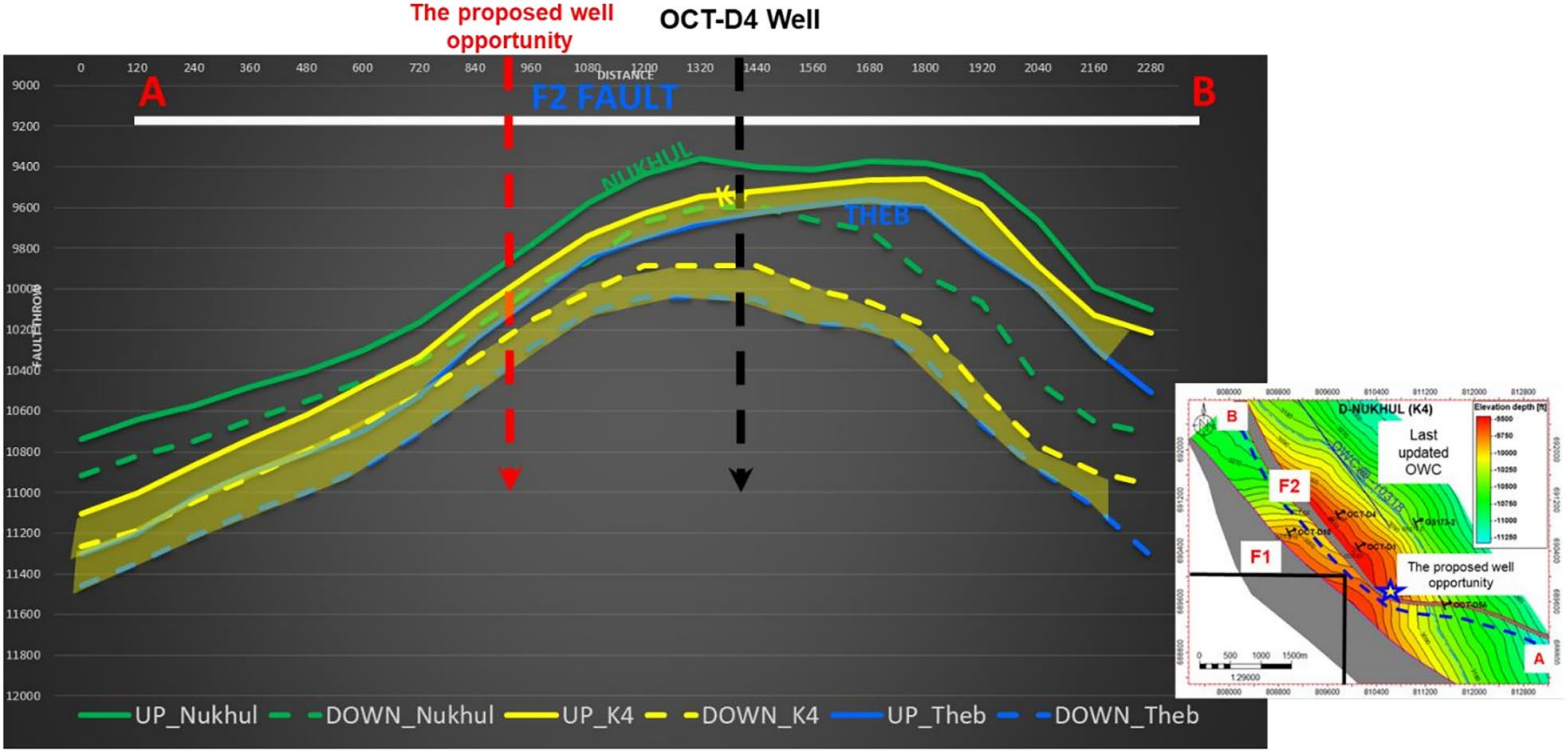
Fault seal analysis across F2 fault to show the up and down thrown juxtaposition relationship to confirm reservoir-sealing character, where the dashed line is representing the down thrown block, while the solid line is representing the Upthrown block.

Fault seal analysis is very important for the proposed well opportunity because it explains and shows what is adjacent to the target reservoir, especially since the study aims to target a part of the Nukhul Formation, known as the K4 zone. Therefore, it is vital to observe and define accurately what lies adjacent to the target reservoir to ensure that the study has addressed every impact factor on the reservoir and to confirm that there are no unexpected conditions affecting oil accumulation and sealing, particularly since well data confirm the presence of oil. Considering the two faults that bound the study area and mainly define the target well opportunity, the most important fault for the fault seal analysis is the F2 fault. As shown in (Fig. 11), line A–B represents the fault seal profile along the F2 fault; this line illustrates the juxtaposition between the upthrown and downthrown blocks to confirm sealing and reservoir continuity.

Figure 11 shows that the dashed line represents the downthrown block, while the solid lines represent the upthrown block, as indicated in the legend at the base of the figure. The limestone–marl units that make up the majority of the basal portion of the Mheiherrat and Nukhul formations, according to lithological and stratigraphic data, are generally characterized by poor permeability and are probably going to act as efficient seals. The main results from this stage of work are as follows:

1. The K4 in the upthrown block is juxtaposed against the basal part of the Mheiherrat and Nukhul Limestone-marl units in the downthrown block.
2. The fault throw is approximately 250–300 ft at the proposed well opportunity location.

All of these findings suggest that F2 is most likely serving as a lateral seal for the K4 zone, forming a structural trap that is surrounded by faults and has little chance of leaking across them. This lowers uncertainty in forecasting hydrocarbon column continuity and validates the feasibility of the suggested attic well location.

## Discussions

### Depth imaging, seismic–well integration, and structural uncertainty

The updated fault structure (F1–F2) and Miocene horizon geometry (Figs. 5, 6, 7, 8, 9) are based on the depth-migrated seismic volume, which has been calibrated by well control. The use of 3D PSDM in conjunction with well-tied interval velocities is crucial to minimizing structural mis-ties at the reservoir level since velocity heterogeneity and fault-related lateral velocity contrast can bias reflector positioning (Nukhul K1–K4). Time-to-depth conversion errors are within tolerances for prospect ranking, and the velocity model is geologically credible based on the close agreement between interpreted throws (≈250–300 ft at the suggested location) and fault-related missing sections found in correlation (Fig. 6).

However, residual depth uncertainty, as indicated by seismic-to-well tie mis-ties within the velocity-calibrated PSDM dataset should be explicitly incorporated into volumetric ranges and landing-depth contingencies, especially at block-bounding faults where minor depth shifts result in a substantial risk of water cut near the oil–water interface. Best practices include (i) testing alternative anisotropy/gradient terms in the velocity model where evaporites or high-contrast lithologies are present (Belayim–South Gharib), (ii) iterating well–seismic ties with impedance-constrained synthetics over the target interval, and (iii) quantifying structural uncertainty envelopes on maps and sections used for well planning^27,32^. By taking these precautions, sidetrack misplacement due to depth is prevented and attic targets defined by small crest upwarp or throw changes are kept robust under realistic velocity circumstances.

### Fault-related compartmentalization and seal behaviour

The Nukhul reservoir is divided into at least two structural compartments by the identified F1 and F2 faults (Figs. 7, 8, 9). A juxtaposition of K4 in the upthrown block against Mheiherrat–Nukhul limestone in the downthrown block is shown by the correlation-based missing sections and the F2 fault-seal section (Fig. 11). This arrangement is frequently linked to partial across-fault transmissibility reduction. Seal capability is determined by (i) the stratigraphic juxtapositions and (ii) the fault rock qualities arising from deformation in traditional juxtaposition-dominated traps; both must be suitable to support hydrocarbon columns^33,34^. Shale Gouge Ratio-type predictors frequently explain reported across-fault flow behaviour and column heights when clay-rich periods are present in the throw path^35,36^.

The observed production behaviour, together with the fault-bounded compartmentalization evident in the structural maps, despite the absence of detailed clay smear metrics, point to a mixed sealing architecture: along-fault or fracture-assisted baffles that locally improve connectivity within the upthrown panel coexist with reduced transmissibility across F2 at K4 (supporting attic trapping). Particularly in cases where the fault rock contains carbonate units, this hybrid behaviour is consistent with recent fault-zone flow syntheses that demonstrate high anisotropy—better along-fault migration potential compared to a cross-fault sealing^37,38^. Operationally, this means that step-rate tests should be created to bind across-fault transmissibility multipliers utilized in simulation, and pressure surveillance should focus on differential build-up/decline across F2.

### Reservoir architecture, fluid distribution, and production risk

Together with seismic facies and well control, the stratigraphic division of the Nukhul into K1–K4 members reveals a layered, heterolithic structure comprising channelized and bar-form sand bodies surrounded by more carbonate-rich or shalier levels. Lighter oils usually occupy structurally higher panels and heavier oils pool downdip or in isolated compartments, which is consistent with the observed 14–39°API range in the field. In these settings, compartment boundaries and subtle structural relief exert first-order control over fluid contacts and API gravity trends.

This architecture presents two competing risks from a production perspective: (i) under-drainage of high-relief attic pods where crestal sands are isolated by limestone–marl sealing units or low-permeability fault rock, and (ii) premature water breakthrough where wells are landed too low relative to the local OWC or across-fault spill points. By enhancing depth control at F2 and defining crest-adjacent lobes for selective completions, the revised structural model reduces both risks. We suggest using (a) PLT-calibrated net-to-gross and facies from cored wells (GS173-2, OCT-D4, OCT-D5A), (b) along-fault versus across-fault transmissibility multipliers derived from carbonate–marl fault-rock analogs, and (c) pressure-transient diagnostics across compartments to validate seal assumptions before full-bore infill, in order to translate structural understanding into reserves. It has been demonstrated that this methodology, which is typical in contemporary integrated reservoir studies, lowers forecast variance and reduces the likelihood of unexpected water-handling requirements in the early stages of attic producers’ lives^27,35,37^.

### Implications for development strategy: attic targeting, well spacing, and low-cost incremental recovery

The most recent data from multiple disciplines was integrated with earlier work to create the updated structural model. The development plan accomplished in this study will benefit from the updated structural model’s assistance in locating attic spaces and undrilled areas. Therefore, based on the most recent structural model scenario, the main conclusion is that there are multiple target opportunities along the updated structure contour maps, and these will be clarified in the conclusions and recommendations section of this paper. The study’s attic areas will boost the oil field’s oil reserves and production. The structural model will aid in delineating and understanding oil–water contact extension, reservoir extension, and reservoir nature, including channels, bars, and levees. Increased oil recovery and successful development plans result from comprehensive areal structural understanding.

As a result of the updated structural model and geological cross sections, the study can reveal faults, block boundaries, and support detection of source rocks and reservoir juxtaposition, as well as characterize sealing types to support geomechanics analyses, which are crucial for comprehending trapping, oil migration pathways, and accumulation in the October oil field.

### Recommendations

**Fig. 12:**
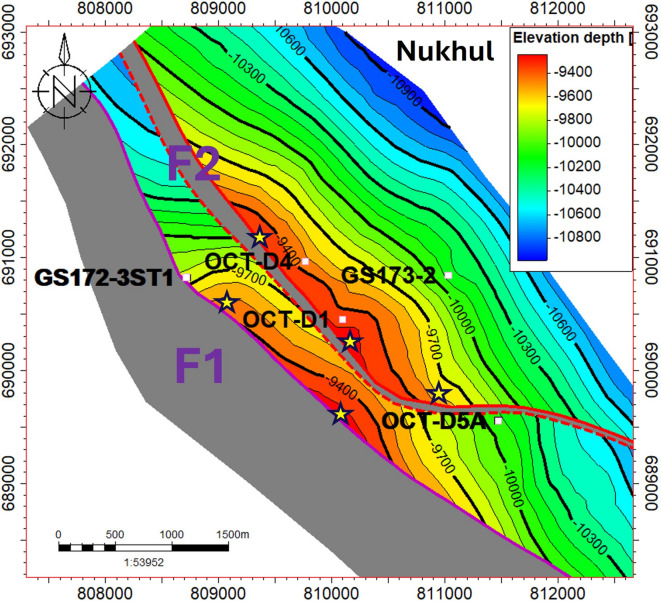
Updated structure contour map highlighting proposed opportunities (stars) targeting attic oil and undrilled compartments.

The study recommends, and emphasizes the importance of taking into consideration, that there are different proposed areas that need to be developed, as shown in (Fig. 12). The stars on the map indicate areas that have not been penetrated in both compartments at the Nukhul level and where well spacing between wells is too large. Under the guidance of fault-seal diagnostics and high-resolution structure maps, sidetracks and short-reach crestal producers provide a tried-and-true, capital-efficient route to incremental recovery in mature, fractured rift settings. Therefore, it is recommended to drill the proposed locations as part of an oil-production acceleration strategy, leveraging the potential of attic locations to increase oil production and meet industry targets. As demonstrated by industry experience, strategically positioned attic wells can add thousands of BOPD at low unit development costs, especially when connected to existing slots and infrastructure. However, the success of these wells depends on (i) accurate depth control ("Depth imaging, seismic–well integration, and structural uncertainty" section), (ii) confirming the integrity of the across-fault seal ("Fault-related compartmentalization and seal behaviour" section), and (iii) having a surveillance plan (offset pressure, rate–transient analysis, and water cut tracking) to prevent encroachment from downdip aquifers.

Encourage a phased approach, which includes updating the static/dynamic models with measured across-fault transmissibility, acquiring DFITs and mini-interference tests across F2, and drilling new wells or sidetracks with contingency landing depths to target indicated areas (Fig. 12). A subsequent pattern of infills can be carried out to speed recovery while preserving pressure support via selective injection in downdip panels if pilots verify the anticipated drawdown and low cross-flow. This phased strategy aligns with best practices documented globally for physically segmented reservoirs and attic oil exploitation^27,35,37^.

Additionally, the new proposed wells are expected to have lower water saturation due to their attic locations compared to current wells. At the same time, these wells may replace existing wells that have mechanical issues or oil production rates lower than expected. This approach will save substantial costs, as side-tracking from current wells is much cheaper than drilling from new slots. This means more oil production by increasing the daily oil rate at a low cost while also increasing oil reserves. Preliminary estimates suggest that this could add approximately 3,000–4,000 barrels of oil per day (BOPD).

## Conclusions

- The 3D structure model in the October Oil Field was thoroughly updated and improved using the flow chart that was created by integrating data from newly drilled and seismic wells, including E-Logs, stratigraphic correlations, and dynamic data.
- The updated structural model indicates a notable influence on reservoir fluid distribution and API gravity, suggesting that lighter fluids tend to occur in up-dip structural positions, as reflected by API values ranging from 14 to 39° from DST tests. This outcome supports the interpretive linkage between structural configuration and variations in hydrocarbon phase and quality.
- The developed model suggests an improved capability to delineate the geometry, extension, and spatial relationships of major faults (F1 and F2), including fault-bounded compartmentalization, limestone–marl seal continuity, and block boundary definition. These results support dividing the area into distinct structural blocks so that each compartment may be addressed with a tailored reservoir management strategy.
- The structural interpretation supports development planning for the targeted area by identifying potential attic locations that may enable infill drilling opportunities, selective completions in attic zones, and reduction of water-breakthrough risk.
- The updated model indicates an improved understanding of the location and continuity of the oil–water contact (OWC) across structural compartments, which may reduce uncertainty in reserve estimation. It also supports a more lithologically consistent fault-seal analysis by evaluating sealing potential and trapping behavior using carbonate–marl juxtaposition criteria.

## Data Availability

Datasets used in this research are available with the corresponding author email: (mostafakhatab@gstd.sci.cu.edu.eg) upon reasonable request.
